# Reporting of Trial Registration Numbers in Publications of Vaccine Randomized Clinical Trials

**DOI:** 10.1001/jamanetworkopen.2025.2276

**Published:** 2025-03-28

**Authors:** Naheemot O. Sule, Yang Zhang, Nicole E. Basta

**Affiliations:** 1 Department of Epidemiology, Biostatistics, and Occupational Health, McGill University, Montreal, Quebec, Canada

## Abstract

This cross-sectional study assesses reporting of trial registration numbers in vaccine-related randomized clinical trial publications and evaluates whether trial registration was prospective.

## Introduction

In vaccine development and testing, randomized clinical trials (RCTs) provide critical evidence on safety, efficacy, and immunogenicity. However, there are concerns about selective reporting of RCT results.^1,2^ The World Health Organization (WHO) and International Committee of Medical Journal Editors (ICMJE) recommend that investigators prospectively register trials and include trial registration numbers (TRNs) in publications to improve transparency in reporting results.^3,4^ Given public health implications of vaccine RCTs, transparency is crucial for building trust in research. Therefore, in this cross-sectional study, we assessed TRN reporting in vaccine-related RCT publications and whether trial registration was prospective.

## Methods

In November 2023, we identified vaccine-related RCT publications indexed on PubMed from 2017 to 2022 by searching: PubMed[Database] AND Vaccines[MeSH] AND 2017:2022[Date – Publication] on CENTRAL. Identified publications were screened for TRNs. We automated the process of searching for, identifying, and extracting TRNs from the citation metadata and full text (eMethods in Supplement 1).^5^ Publications without TRNs were manually screened to confirm eligibility and absence of TRNs. This study followed the STROBE reporting guideline. Institutional review was not required according to both the McGill University institutional review board and the Common Rule as all data are publicly available.

We determined the proportion of publications reporting TRNs and reviewed registry records to assess prospective registration. We used bivariate logistic regression to analyze associations between TRN reporting and publication characteristics from metadata, including ICMJE connection of the journal, publication year, language, and first author’s affiliated country. A 2-sided *P* < .05 was considered statistically significant. Analyses were conducted using R, version 4.1.0.

## Results

Our search identified 2626 publications. We analyzed 2174 after removing 39 duplicates, 3 unretrievable manuscripts, and 410 nonhuman or non-RCT publications (eFigure in Supplement 1). We found that 90.9% (n = 1976) reported at least 1 TRN; 55.7% had TRNs in citation metadata, 8.7% within abstracts, and 26.5% only in full text. Annual reporting from 2017 to 2022 ranged from 89.2% to 91.6% (Table 1).

**Table 1.** Association Between Characteristics of 2174 Vaccine-Related Randomized Clinical Trial Publications From 2017 to 2022 and TRN Reporting

Characteristic	No. of records		Bivariate logistic regression	
	Total No.	With TRNs, No. (%)	OR (95% CI)	*P* value
Overall	2174	1976 (90.9)	NA	NA
ICMJE group of journal				
ICMJE member	415	407 (98.1)	1 [Reference]	NA
Follow recommendations	1046	960 (91.8)	0.22 (0.10-0.43)	<.001
Nonmember	713	609 (85.4)	0.12 (0.05-0.22)	<.001
First author’s country income group				
HIC	1518	1406 (92.6)	1 [Reference]	NA
UMIC	391	332 (84.9)	0.45 (0.32-0.63)	<.001
LMIC	188	164 (87.2)	0.54 (0.35-0.89)	.01
LIC	77	74 (96.1)	1.96 (0.72-8.10)	.26
Publication year				
2017	313	286 (91.4)	1 [Reference]	NA
2018	292	267 (91.4)	1.01 (0.57-1.79)	.98
2019	360	327 (90.8)	0.94 (0.55-1.59)	.81
2020	344	307 (89.2)	0.78 (0.46-1.31)	.36
2021	413	375 (90.8)	0.93 (0.55-1.56)	.79
2022	452	414 (91.6)	1.03 (0.61-1.72)	.91
Language of publication				
English	2157	1973 (91.2)	1 [Reference]	NA
Non-English	17	3 (12.5)	0.02 (0.005-0.06)	<.001

Abbreviations: HIC, high-income country; ICMJE, International Committee of Medical Journal Editors; LIC, low-income country; LMIC, lower middle-income country; NA, not applicable; OR, odds ratio; TRN, trial registration number; UMIC, upper middle-income country.

We extracted 2521 valid TRNs and 1816 unique TRNs. Only 63.2% of corresponding trials were prospectively registered (Table 2). The proportion of prospectively registered vaccine-related RCTs from a given trial registry ranged from 0% to 94.1% (Table 2). Among 668 trials not prospectively registered, 60.3% were registered within 90 days of enrollment beginning, 17.2% between more than 3 months and 1 year, 14.5% between more than 1 year and 3 years, and 7.9% more than 3 years after enrollment.

**Table 2.** Prospective Registration Status of the 1816 Unique Trial Registration Numbers Extracted From Vaccine-Related Randomized Clinical Trial Publications

Trial registry	Trials extracted per registry, total No.	Trials prospectively registered, No. (%)
ClinicalTrials.gov	1432	881 (61.5)
EU Clinical Trials Register (EUCTR/EudraCT)	85	80 (94.1)
International Standard Randomized Controlled Trial Number–UK (ISRCTN)	58	33 (56.9)
Clinical Trials Registry–India (CTRI)	49	40 (81.6)
Australian New Zealand Clinical Trials Registry (ANZCTR)	43	26 (60.5)
Japan Primary Registries Network (JPRN: jRCT; JAPIC-CTI; UMIN; JMACCT)	40	24 (60.0)
Chinese Clinical Trial Register (ChiCTR)	29	16 (55.2)
Pan African Clinical Trial Registry (PACTR)	21	16 (76.2)
Iranian Registry of Clinical Trials (IRCT)	15	7 (46.7)
Thai Clinical Trials Register (TCTR)	13	7 (53.9)
The Netherlands National Trial Register (NTR)	11	9 (81.8)
Brazilian Clinical Trials Registry (ReBec)	6	3 (50.0)
Cuban Public Registry of Clinical Trials (RPCEC)	6	3 (50.0)
Peruvian Clinical Trials Registry (REPEC)	3	2 (66.7)
Clinical Research Information Service (CRiS), Republic of Korea	3	0
German Clinical Trials Register (DRKS)	2	1 (50.0)
Sri Lanka Clinical Trials Registry (SLCTR)	0	0
Total	1816	1148 (63.2)

Abbreviations: JAPIC-CTI, Japan Pharmaceutical Information Center Clinical Trials Information; JMACCT, Japan Medical Association Center for Clinical Trials; jRCT, Japan Registry of Clinical Trials; UMIN, University hospital Medical Information Network.

Compared with publications in ICMJE member journals, those in nonmember journals (odds ratio [OR], 0.12 [95% CI, 0.05-0.22]) and those in journals pledging to follow ICMJE recommendations (OR, 0.22 [95% CI, 0.10-0.43]) had lower odds of reporting TRNs (Table 1). In addition, publications with first authors affiliated with upper middle- and lower middle-income countries had lower odds of reporting TRNs (OR, 0.45 [95% CI, 0.32-0.63] and 0.54 [95% CI, 0.35-0.89], respectively) than those with first authors with high-income country affiliations. Non-English publications also had lower odds of reporting TRNs (OR, 0.02 [95% CI, 0.005-0.06]), although this assessment is limited by the small sample (n = 17). Publication year was not significantly associated with reporting TRNs.

## Discussion

We found that 90.9% of vaccine-related RCTs published from 2017 to 2022 reported TRNs, but only 63.2% of trials were prospectively registered. As noted by the WHO and ICMJE, improving prospective trial registration and TRN reporting remains critical to improving transparency in RCT research.^3,4^

To our knowledge, our study is the largest assessment of TRN reporting and prospective trial registration for vaccine RCTs conducted. Strengths include the large sample of vaccine RCTs published over multiple years and the tailored algorithm we developed and tested to automate extraction of TRNs. Limitations include that our algorithm was not designed or optimized to extract trial characteristics such as sample size, vaccine target, and whether the manuscript was a primary or secondary report. Future studies could build on this study by investigating these factors. Identifying strategies for improving TRN reporting and prospective registration practices remains a priority.

## Notes

Supplement 1. **eMethods.** Automated Extraction of Trial Registration Numbers (TRNs) **eFigure.** Identification of Eligible Vaccine Trial Publications and the Process of Identifying and Extracting Trial Registration Numbers (TRNs)
eReferences.

Supplement 2.
Data Sharing Statement
