# Supplementary material for: Reporting of Trial Registration Numbers in Publications of Vaccine Randomized Clinical Trials
Source: JAMA Netw Open. 2025 Mar 28;8(3):e252276. doi: 10.1001/jamanetworkopen.2025.2276 (PMC11953748; doi:10.1001/jamanetworkopen.2025.2276)
Supplement: Supplement 1. — eMethods. Automated Extraction of Trial Registration Numbers (TRNs) eFigure. Identification of Eligible Vaccine Trial Publications and the Process of Identifying and Extracting Trial Registration Numbers (TRNs) eReferences. [file jamanetwopen-e252276-s001.pdf]

Supplementary Online Content

Sule NO, Zhang Y, Basta NE. Reporting of trial registration numbers in publications of vaccine randomized clinical trials. *JAMA Netw Open.* 2025;8(3):e252276. doi:10.1001/jamanetworkopen.2025.2276

eMethods. Automated Extraction of Trial Registration Numbers (TRNs)
eFigure. Identification of Eligible Vaccine Trial Publications and the Process of Identifying and Extracting Trial Registration Numbers (TRNs)
eReferences.

This supplementary material has been provided by the authors to give readers additional information about their work.

© 2025 Sule NO et al. *JAMA Network Open.*

eMethods. Automated Extraction of Trial Registration Numbers (TRNs)

We extracted trial registration number (TRNs) and corresponding trial registry names in three steps. First, we used R to screen the PubMed metadata of each citation record to identify TRNs and the corresponding trial registry that issued the TRN[1]. PubMed's metadata field <DatabankName> stores the name of the trial registry and the field <AccessionNumber> stores the TRN for those publications reporting the results of an RCT. The TRN information is only available in the metadata if the publishing journal provided this information, or a Pubmed staff manually copies and pastes it from the abstract or full manuscript[2-4]. We used R to extract both the trial registry name and the associated TRNs when available.

Next, we focused on records without TRNs in the <AccessionNumber> metadata element by searching the <Abstract> metadata field which contains text of the publication’s abstract. To search this text field systematically, we developed an R algorithm that uses advanced text search patterns to screen for TRNs within the abstract and extract them. There are 20 trial registries indexed by PubMed into their metadata fields and recognized as primary or partner registries in the WHO registry network[3, 5, 6] and we screened each abstract to determine whether trials from any of these 20 trial registries were reported. We developed these search patterns using the TRN format commonly used by each trial registry. For example, in the case of 'NCT0123456,' a Clinicaltrials.gov number, the pattern we developed searched and extracted for all occurrences of NCT alphanumeric combinations such as NCT0123456, nct0123456, NCT: 0123456, NCT 0123456 and NCT#: 0123456. This principle was applied to the TRN formats of all the 20 trial registries we screened.

Finally, for records without TRNs in the any of these 3 metadata fields, we retrieved the full-text of the publications, and PaperPile (Paperpile LLC, 2024) referencing software was used to download and compile the full-text publications[7]. The text of these publications were then uploaded into R. Using the algorithm we developed, we screened and extracted TRNs from the full-text publications.

© 2025 Sule NO et al. *JAMA Network Open.*

eFigure. Identification of Eligible Vaccine Trial Publications and the Process of Identifying and Extracting Trial Registration Numbers (TRNs)

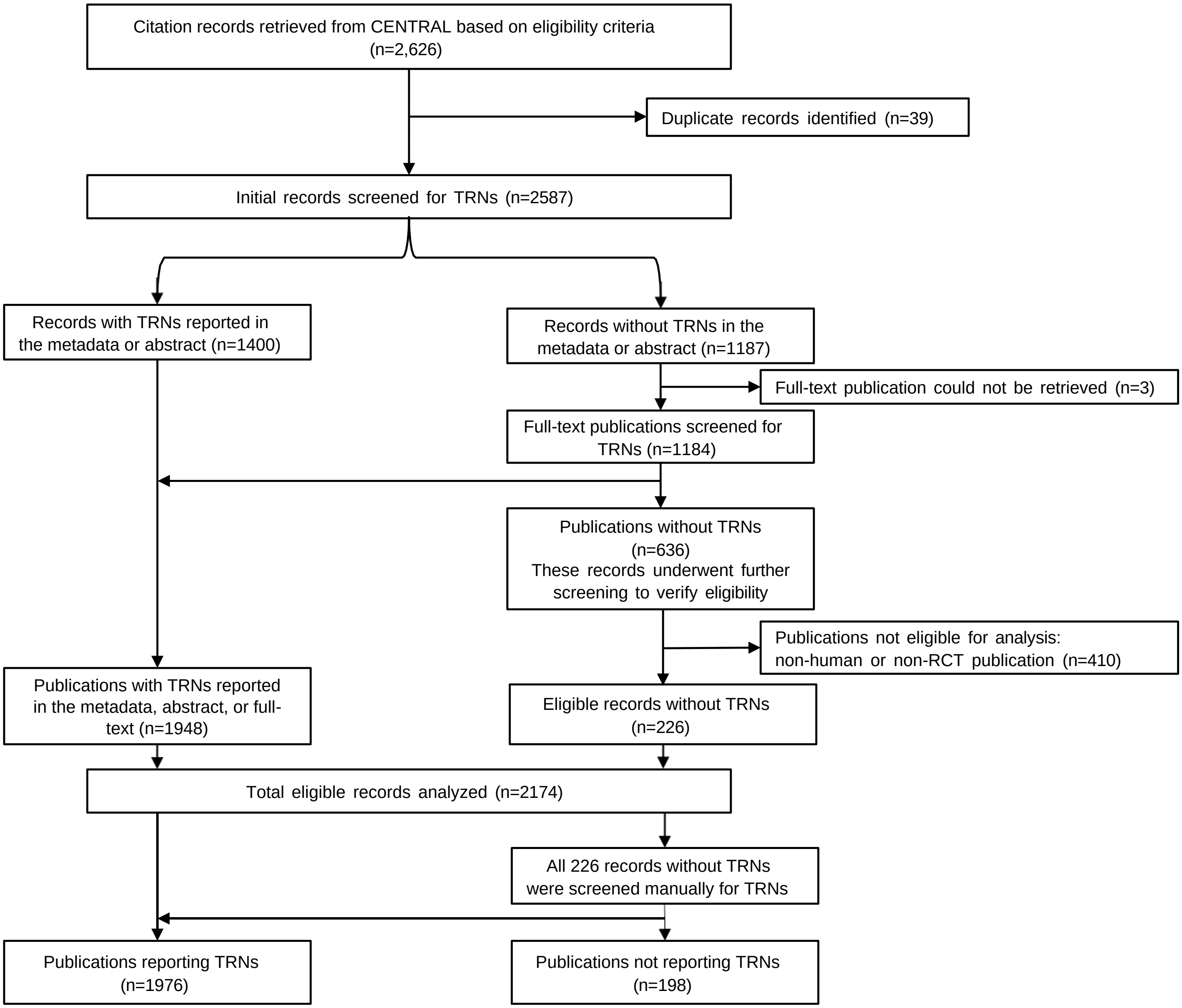

© 2025 Sule NO et al. *JAMA Network Open.*

eReferences.

1. Fantini D. Retrieving and Processing PubMed Records using easyPubMed, https://cran.r-project.org/web/packages/easyPubMed/vignettes/getting_started_with_easyPubMed.html (accessed September 15, 2023).
2. MEDLINE®PubMed® XML Element Descriptions and their Attributes. 2018, https://www.nlm.nih.gov/bsd/licensee/elements_descriptions.html (accessed February 22, 2024).
3. MEDLINE Databank Sources, https://www.nlm.nih.gov/bsd/medline_databank_source.html (accessed February 22, 2024.).
4. Salholz-Hillel M, Strech D and Carlisle BG. Results publications are inadequately linked to trial registrations: An automated pipeline and evaluation of German university medical centers. *Clinical Trials* 2022; 19: 337-346. DOI: 10.1177/17407745221087456.
5. Primary registries in the WHO registry network, https://www.who.int/clinical-trials-registry-platform/network/primary-registries (accessed February 26, 2024).
6. WHO Registry Network Partner registries, https://www.who.int/clinical-trials-registry-platform/network/partner-registries (accessed February 26, 2024).
7. Paperpile: The no-fuss reference manager for the web [Paperpile Website]. 2023. https://paperpile.com/?welcome (accessed February, 20 2024).
8. ICMJE: Journals stating that they follow the ICMJE Recommendations, https://www.icmje.org/journals-following-the-icmje-recommendations/ (accessed February 20, 2024).

© 2025 Sule NO et al. *JAMA Network Open.*
