# Supplementary material for: Reporting of Trial Registration Numbers in Publications of Vaccine Randomized Clinical Trials
Source: JAMA Netw Open. 2025 Mar 28;8(3):e252276. doi: 10.1001/jamanetworkopen.2025.2276 (PMC11953748; doi:10.1001/jamanetworkopen.2025.2276)
Supplement: Supplement 2. — Data Sharing Statement [file jamanetwopen-e252276-s002.pdf]

Data Sharing Statement

Sule. Reporting of Trial Registration Numbers in Publications of Vaccine Randomized Clinical Trials. *JAMA Netw Open*. Published March 28, 2025. doi:10.1001/jamanetworkopen.2025.2276

Data
Data available: Yes
Data types: Data (not involving human participants)
How to access data: All data used in this analysis are publicly available and can be accessed via www.cochranelibrary.com/central/about-central according to the methods described.
When available: With publication

Supporting Documents
Document types: None

Additional Information
Who can access the data: Anyone-All data used in this analysis are publicly available
Types of analyses: All data used in this analysis are publicly available
Mechanisms of data availability: All data used in this analysis are publicly available
